# hMPV Outbreaks: Worldwide Implications of a Re-Emerging Respiratory Pathogen

**DOI:** 10.3390/microorganisms13071508

**Published:** 2025-06-27

**Authors:** Alexandra Lianou, Andreas G. Tsantes, Petros Ioannou, Efstathia-Danai Bikouli, Anastasia Batsiou, Aggeliki Kokkinou, Kostantina A. Tsante, Dionysios Tsilidis, Maria Lampridou, Nicoletta Iacovidou, Rozeta Sokou

**Affiliations:** 1 Neonatal Intensive Care Unit, “Agios Panteleimon” General Hospital of Nikea, 18454 Piraeus, Greece; alexlianou95@gmail.com (A.L.); abatsiou@gmail.com (A.B.); angie_skala@hotmail.com (A.K.); marialampridou1@hotmail.com (M.L.); 2 Microbiology Department, “Saint Savvas” Oncology Hospital, 11522 Athens, Greece; 3 Laboratory of Haematology and Blood Bank Unit, “Attiko” Hospital, School of Medicine, National and Kapodistrian University of Athens, 12462 Athens, Greece; ktsante@yahoo.com; 4 School of Medicine, University of Crete, 71003 Heraklion, Greece; 5 Neonatal Intensive Care Unit, General and Maternity Hospital “Helena Venizelou”, 11521 Athens, Greece; danai_mp89@yahoo.gr; 6 Department of Medicine, National and Kapodistrian University of Athens, 11527 Athens, Greece; dtsilides@gmail.com; 7 Neonatal Department, National and Kapodistrian University of Athens, Aretaieio Hospital, 11528 Athens, Greece; niciac58@gmail.com

**Keywords:** human metapneumovirus, respiratory infections, public health surveillance, global health, pediatric, perinatal, neonates, vaccines, pregnancy

## Abstract

Human metapneumovirus (hMPV), a member of the *Pneumoviridae* subfamily, has emerged as a significant etiological agent of acute respiratory tract infections across diverse age groups, particularly affecting infants, the elderly, and immunocompromised individuals. Since its initial identification in 2001, hMPV has been recognized globally for its seasonal circulation pattern, predominantly in late winter and spring. hMPV is a leading etiological agent, accounting for approximately 5% to 10% of hospitalizations among pediatric patients with acute respiratory tract infections. hMPV infection can result in severe bronchiolitis and pneumonia, particularly in young children, with clinical manifestations often indistinguishable from those caused by human RSV. Primary hMPV infection typically occurs during early childhood; however, re-infections are frequent and may occur throughout an individual’s lifetime. hMPV is an enveloped, negative-sense RNA virus transmitted through respiratory droplets and aerosols, with a 3–5-day incubation period. The host immune response is marked by elevated pro-inflammatory cytokines, which contribute to disease severity. Advances in molecular diagnostics, particularly reverse transcription–quantitative polymerase chain reaction (RT-qPCR) and metagenomic next-generation sequencing (mNGS), have improved detection accuracy and efficiency. Despite these advancements, treatment remains largely supportive, as no specific antiviral therapy has yet been approved. Promising developments in vaccine research, including mRNA-based candidates, are currently undergoing clinical evaluation. This review synthesizes current knowledge on hMPV, highlighting its virological, epidemiological, and clinical characteristics, along with diagnostic advancements and emerging therapeutic strategies, while underscoring the critical role of continued research and sustained preventive measures—including vaccines, monoclonal antibodies, and non-pharmaceutical interventions—in mitigating the global burden of hMPV-related disease.

## 1. Introduction

Human metapneumovirus (hMPV) is a negative-sense, single-stranded RNA virus classified within the *Metapneumovirus* genus of the *Pneumoviridae* family. First isolated in the Netherlands in 2001, hMPV has since been identified worldwide as a prominent cause of acute respiratory tract infections [1]. Its genome, approximately 13 kilobases in length, encodes several structural and non-structural proteins essential for viral replication and host interaction [2,3]. The virus is primarily transmitted through respiratory droplets or direct contact with infected individuals, with an incubation period typically ranging from three to six days [4].

hMPV is a globally prevalent respiratory virus primarily affecting children under five years of age [5,6], with documented outbreaks reported across diverse geographical regions including North and South America, Europe, and Asia. While infection rates are highest in infants and young children, older adults—particularly those aged 65 and above—also experience significant morbidity, especially when hospitalized with underlying comorbidities [7]. Surveillance data estimate approximately 473,000 hMPV-associated hospital admissions worldwide among older adults in 2019, highlighting its considerable clinical burden across both pediatric and elderly populations in various healthcare settings [7].

The seasonality of hMPV is well characterized in temperate climates, where incidence peaks during the late winter to early spring months, typically coinciding with respiratory syncytial virus (RSV) activity [4,5]. In tropical and subtropical regions, such as India and Southeast Asia, hMPV circulation is influenced by local climatic conditions, often peaking during or shortly after the monsoon season [8]. These distinct epidemiological patterns underscore the influence of environmental factors on viral transmission dynamics across different global regions. Following a global decline in hMPV circulation during the COVID-19 pandemic due to public health interventions, a post-pandemic resurgence has emerged, often with atypical seasonal patterns. This re-emergence is likely driven by reduced population immunity and co-circulation with other respiratory viruses [5,9,10]. Recent data also indicate altered transmission dynamics, including higher incidence in older children and increased household clustering [5,11].

Clinically, hMPV infections present with a spectrum of respiratory symptoms ranging from mild upper respiratory tract illness to severe lower respiratory tract disease, including bronchiolitis and pneumonia [12,13]. While healthy adults often experience mild symptoms, infants, older adults, and immunocompromised individuals are at higher risk for severe outcomes [7]. Beyond its independent pathogenicity, hMPV is frequently implicated in co-infections with other respiratory viruses, including influenza, RSV, and SARS-CoV-2, which may exacerbate clinical outcomes [14]. Despite its clinical significance, there are currently no licensed antiviral treatments or vaccines available for hMPV, and management remains supportive [15,16]. This underscores the need for ongoing research into the virus’s epidemiology, pathogenesis, and potential treatment strategies.

This review provides a comprehensive overview of the current knowledge on hMPV infection, focusing on its virological characteristics, epidemiological trends, and clinical manifestations, while synthesizing recent data on its global burden, seasonal trends, age-specific risks, and geographic variability. Furthermore, the review critically examines advances in diagnostic methods and emerging therapeutic strategies, including vaccine development, monoclonal antibody therapies, and non-pharmaceutical interventions, aiming to highlight future research priorities and public health strategies to mitigate the impact of hMPV-associated disease worldwide.

## 2. Pathophysiology

### 2.1. Structure and Genotypes

hMPV is an encircled, viral agent of approximately 209 nanometer in diameter [2]. The hMPV genome is composed of a negative, single-stranded, and non-segmented RNA sequence of around 13 kilobases in length. The hMPV genome hosts eight genes which encode nine structural proteins, with a discrete amino acid chain identity of 3′-N-P-M-F-M2(-1/-2)-SH-G-L-5′ [2,3]. Each open reading frame (ORF) is surrounded by untranslated regions that play a regulatory role in terminating and reinitiating transcription. This regulatory mechanism leads to a transcriptional gradient, with mRNA abundance progressively decreasing from the 3′ end toward the 5′ end of the viral genome [17].

**Figure 1:**
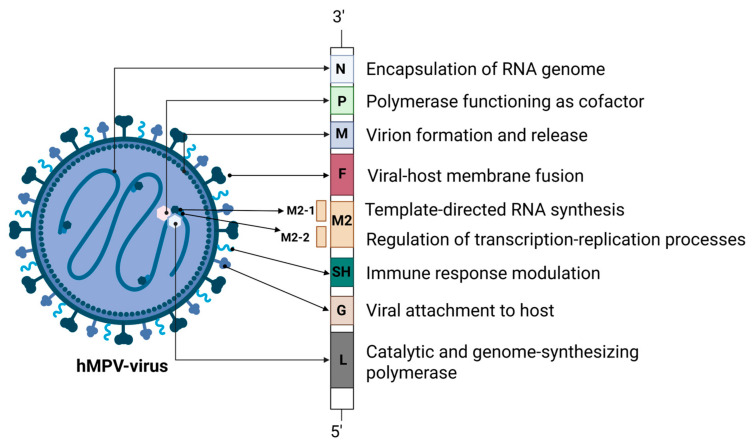
Overview of hMPV proteins: structure and functional characteristics. hMPV: human metapneumovirus; RNA: ribonucleic acid; SH: small hydrophobic protein. Created in BioRender. L., A. (accessed on 30 April 2025) https://BioRender.com/zj1pa3c.

The N protein, a 43.5 kDa molecular weight nucleoprotein, contributes to the encapsidation of the single stranded RNA genome, forming a nucleoprotein nexus which facilitates the protection of the hMPV genome from degradation by host nucleases [18]. The P protein, is a phosphoprotein of 32.4 kDa and is part of the viral RNA polymerase complex [19]. This protein acts as an essential cofactor, enhancing the structural stability of the viral polymerase L and promoting the efficient assembly of the ribonucleoprotein (RNP) complex required for viral RNA replication [15,16]. It plays a crucial role in assisting the interaction of the L protein with the viral N–RNA protein complex, by binding to both N and L at the same time, ultimately allowing the virus to replicate and transcribe its genome into new viral particles [20,21]. The large polymerase (L) protein (230.6 kDa) contains zinc-binding sites and exhibits multifunctional catalytic activity, playing a key role in viral genome synthesis alongside various cofactors [22]. The RNA genome is closely associated with the viral proteins N, P, L, M2-1, and M2-2, which together constitute the nucleocapsid structure. The nucleocapsid is boarded by the matrix (M) protein, which lines the inner surface of the viral lipid envelope. The M protein is essential for the assembly of the newly formed virus particles and participates in the virion budding process from the host cells, via interacting with various cellular and viral elements [23]. The fusion protein (F), a protein of 58.4 kDa, plays a major role in the invasion of the virus to the host by assisting host cell–virus attachment and the following fusion of viral and cellular membranes, thereby enabling the release of the viral genome into the host cell cytoplasm. The hMPV F glycoprotein is a primary antigenic component and a key focus of the immune system’s neutralizing antibody response [24]. The M2 protein gene contains two open reading frames that enable the expression of the M2-1 (21.2 kDa) and M2-2 (8.1 kDa) proteins. These proteins regulate RNA polymerase activity and are involved in the modulation of the host immune response. In particular, the highly conserved M2-1 protein acts as a zinc-binding transcription anti-terminator and also attaches to mRNA, acting protectively against premature termination [25]. The small hydrophobic (SH) protein (20.9 kDa) modulates the innate immune response by inhibiting the activation of nuclear factor-κB (NF-κB) and interferon (IFN) signaling. It also interferes in the regulation of the fusogenicity of the F protein [26]. The glycoprotein (G) (25.7 kDa) binds to cellular glycosaminoglycans and facilitates viral attachment. It also suppresses the IFN-I response and promotes neutrophil recruitment by enhancing the secretion of chemotactic factors such as C-X-C Motif Chemokine Ligand 2 (CXCL2), C-C Motif Chemokine Ligand 3 (CCL3), CCL4, IL-17, and tumor necrosis factor a (TNF-a) [27] (Figure 1).

Whole-genome sequencing has revealed that hMPV comprises two primary genotypes, designated A and B. These genotypes are further classified into sublineages—A1, A2, B1, and B2—based on genetic variability in the viral surface glycoproteins, specifically the attachment (G) and fusion (F) proteins. The subgroup A2 is further subdivided into A2a and A2b. Notably, a study has identified a strain that clusters within genotype A but does not align with either the A1 or A2 subgroups, suggesting the potential emergence of a novel subgroup within the A genotype [28].

### 2.2. Immunopathology

The hMPV virus is capable of rapidly disseminating throughout the respiratory tract following its initial accumulation in the nasopharyngeal mucosa. hMPV viral particles can display a morphological transition among spherical and filamentous structures and are enveloped by a lipid bilayer membrane adorned with glycoprotein structures measuring around 13 to 17 nanometers [12]. The hMPV genome contains approximately eight genes which encode nine unique proteins that facilitate viral penetration into host cells. Human cells are enveloped by a dense layer of glycoconjugates involved in key homeostatic functions. Pathogens such as hMPV have adapted to target this glycan-rich surface for their initial binding and entry [29]. The wide variety of glycans, characterized by variations in chain length and core structure, creates a heterogeneous environment within the upper and lower respiratory tract, and these glycans can be identified as potential glycoreceptors for hMPV recognition [30].

Airway epithelial cells (AECs) are the main sites of hMPV infection, where the virus adheres to the cell surface by binding its glycoprotein to the heparan sulfate molecules present on AECs. The fusion glycoprotein (F) enables hMPV entry into the host cell by binding to integrins on the cell surface, initiating transmembrane penetration of the viral genetic material with the assistance of the accessory glycoprotein (G) [31]. Upon entering the host cell cytoplasm, the viral nucleocapsid undergoes replication. RNA polymerase converts viral negative-sense RNA into positive-sense mRNA, enabling viral protein synthesis. Viral glycoproteins are then processed through the Golgi apparatus and accumulated at the cell membrane for viral particle assembly. Once the protein levels reach a set level, RNA polymerase replicates the genome into positive-sense RNA, which serves as a template for synthesizing new negative-sense genomic RNA to be incorporated into nascent viral particles [32]. The M protein aids in the assembly of viral particles, allowing their exocytosis from the host cell through membrane budding [32]. Upon entry into host cells, hMPV is recognized by pattern recognition receptors such as Toll-like receptors (TLRs) and RIG-I-like receptors (RLRs), initiating innate immune signaling pathways. TLRs, including TLR2, TLR4, TLR3, and TLR7/8, detect viral components either at the cell surface or within endosomal compartments, leading to the activation of adaptor molecules like MYD88 and TRIF. This engagement results in the activation of nuclear factor kappa B (NF-κB) and interferon regulatory factors (IRFs), culminating in the transcription of antiviral genes, including type I interferons (IFN-α and IFN-β) and pro-inflammatory cytokines such as IL-1β, IL-2, IL-4, IL-6, and IL-8 [33]. Concurrently, cytoplasmic RLRs, notably RIG-I and *Melanoma Differentiation-Associated Gene 5 (MDA5),* identify viral RNA, leading to the activation of mitochondrial antiviral-signaling protein (MAVS). This interaction further stimulates IRF-3 and IRF-7, which translocate to the nucleus to promote the expression of antiviral proteins and cytokines. These coordinated responses limit viral replication and enhance inflammation, underscoring the complex interplay of immune signaling networks during hMPV infection [33,34].

hMPV infection of the airway epithelium triggers a robust innate immune activation characterized by the stimulation of airway epithelial cells, macrophages, and dendritic cells [33,35]. These cells respond by producing a spectrum of proinflammatory cytokines that are implicated in both antiviral defense mechanisms and the promotion of immunopathological effects. Notably, cytokines such as IL-1β, IL-6, IL-8 (CXCL8), TNF-α, Monocyte Chemoattractant Protein-1 (MCP-1), IL-33, and TSLP exhibit markedly upregulated activity in the context of hMPV exposure [36]. In the context of the inflammatory process, monocytes and lymphocytes are recruited to the airway endothelium. Monocyte migration to sites of infection is mediated by MCP-1, a chemokine that plays a key role in promoting airway inflammatory responses [37]. Pro-inflammatory cytokines, and especially IL-8, further stimulate the release of chemokines, which recruit neutrophils to the lung. While these neutrophils play a role in pathogen clearance, their presence also contributes to tissue damage and sustained inflammation [37]. Collectively, these processes contribute to pulmonary inflammation, leading to the manifestation of respiratory symptoms seen in hMPV infections [38].

Along with the secretion of inflammatory cytokines, predominantly with a type 2 profile, hMPV triggers a rapid immune response that involves a significant innate immune component. Cytokines such as TSLP, IL-25, and IL-33 act as potent inducers of type 2 immune responses in innate immune cells, leading to excessive mucus production and the development of lung allergies [39]. The expression of IL-33 and TSLP is significantly increased during hMPV infection, particularly in children, promoting the T-helper 2 (Th2) immune response which exacerbates the progression of lung damage [40]. While the events induced by such cytokines play a critical role in combating viral infections, they also contribute to severe bronchiolitis and persistent pulmonary complications, predominantly in young children, older adults, and immunocompromised individuals [41].

Preclinical studies in animal models demonstrate that in neonates, the immune response to respiratory viral pathogens is skewed toward a type 2 immunological profile. This is characterized by an elevated secretion of epithelial-derived cytokines, including IL-33, IL-25, and TSLP [42]. These cytokines promote the expansion of type 2 innate lymphoid cells (ILC2s) and Th2, as well as the shift of alveolar macrophages toward an M2-like phenotype. Cytotoxic CD8^+^ T lymphocytes in neonates exhibit a reduced T cell receptor (TCR) affinity, and both CD8^+^ T cells and natural killer (NK) cells demonstrate impaired activity [43,44]. Additionally, germinal center (GC) responses are attenuated, with decreased differentiation of T follicular helper (Tfh) cells and a consequent reduction in immunoglobulin G (IgG) production by GC B cells. Especially for hMPV infection, preclinical data is quite limited. Evidence from neonatal mice models supports a host defense against hMPV similar to RSV models [45]. In patients under three years of age with hMPV infection, nasal secretion samples exhibited a relative enrichment of proteins linked to Th1 immune responses, with no corresponding increase in markers of Th2 activity. However, this Th1-skewed immune profile was not observed in children with a history of prematurity, suggesting a disruption in the typical Th1/Th2 balance in this subgroup [46]. In contrast, a separate study analyzing protein concentrations in nasal secretions reported that infants infected with hMPV exhibited a reduced interferon-gamma IFN-γ to IL-4 ratio—indicative of a Th2-skewed immune response—when compared to those infected with RSV or influenza virus [47]. A study examining nasal immune responses in infants revealed that full-term infants exhibited strong antiviral immune responses—marked by elevated IFNγ, CCL5/Regulated upon Activation, Normal T Cell Expressed and Secreted (RANTES), IL-10, and a high IFNγ/IL-4 ratio—during RSV and hMPV infections. In contrast, premature infants displayed relatively weakened responses, especially to hMPV, with little or no increase in these inflammatory markers. These results suggest that premature infants have an impaired antiviral defense, particularly against hMPV, which may explain their heightened risk for severe illness [48].

## 3. Epidemiological Data

hMPV is a globally circulating respiratory pathogen that primarily affects children under the age of five but also poses significant health risks to older adults and immunocompromised individuals. Across diverse geographical regions, surveillance data consistently show the highest hMPV detection rates in infants and young children, particularly those between 6 months and 4 years of age [5,6,49]. While adults generally show lower positivity rates, those over 65 years old are more likely to experience severe disease outcomes, especially when underlying comorbidities are present. Worldwide data concerning the impact of hMPV on older adults reported a pooled hMPV positivity in hospitalized adults aged ≥65 years with acute respiratory tract infection (ARTI), at 2.46% in high-income countries and 2.01% in low-moderate income countries. This corresponds to an estimated 473,000 hMPV-associated hospital admissions globally in 2019. Incidence and hospitalization rates varied by age, region, and setting, with limited mortality data reported [7].

**Figure 2:**
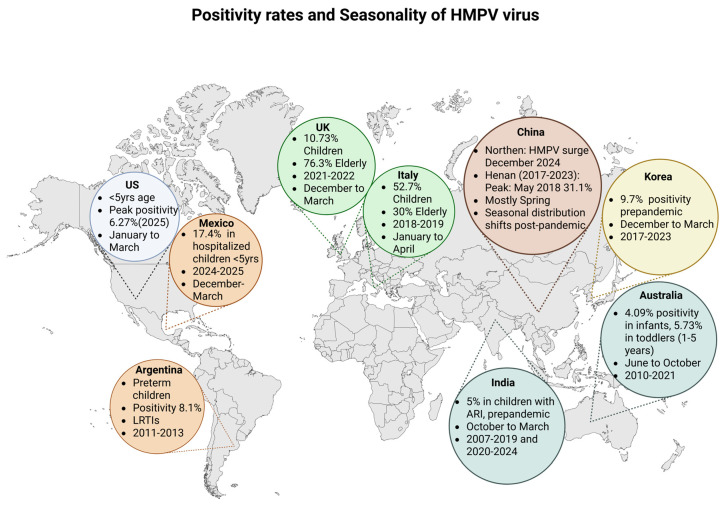
Positivity rates and seasonality of hMPV virus. US: United States; UK: United Kingdom; LRTIs: Lower respiratory tract infections; ARI: acute respiratory infection. Created in BioRender. L., A. (accessed 30 April 2025) https://BioRender.com/39vha56.

hMPV demonstrates a clear global seasonality, typically peaking in late winter to early spring across many temperate regions [4,5,11]. Pre-pandemic, its activity generally followed that of RSV and was concentrated between January and April [5]. However, during the COVID-19 pandemic (2020–2021), a marked decline in hMPV incidence was observed globally, attributed to social distancing and public health measures [5,11,50]. In the post-pandemic period, circulation resumed but with atypical seasonality. For instance some regions, like parts of China and Korea, experienced delayed or shifted peaks into the summer and autumn months, though many have since returned to their pre-pandemic seasonal patterns [5,10,50,51]. Geographically, hMPV affects both hemispheres with varying intensity: high incidence rates are seen in children under five in North and South America, Europe, and Asia, with notable outbreaks reported in the US, China, Mexico, and Argentina [5,9,52,53]. In tropical and subtropical areas, like India and parts of Southeast Asia, hMPV seasonality is influenced by the local climate, often peaking during or after the monsoon season [54]. Post-pandemic epidemiological trends also show increased incidence in older pediatric age groups and more frequent household clustering, suggesting evolving transmission dynamics (Figure 2) [5,6].

### 3.1. United States

**Figure 3:**
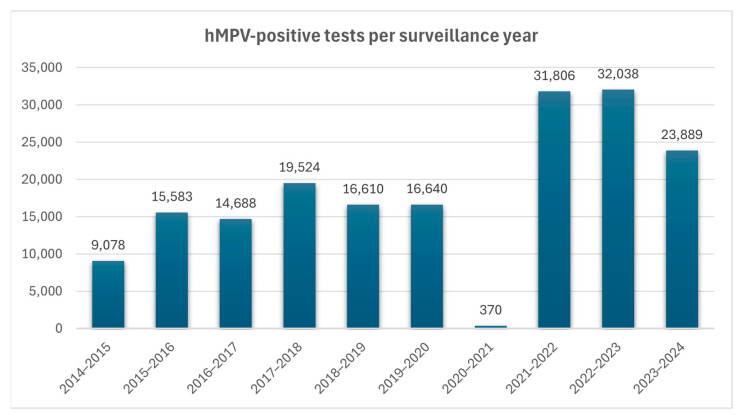
Numbers of hMPV-positive tests per surveillance year. Data collected from Jobe NB, Rose E, Winn AK, Goldstein L, Schneider ZD, Silk BJ. Human Metapneumovirus Seasonality and Co-Circulation with Respiratory Syncytial Virus—United States, 2014–2024 [5].

hMPV remains an endemic virus worldwide, presenting seasonal peaks of disease spread annually. Prior to the pandemic of COVID-19, the seasonal activity of hMPV generally commenced in early January, reaching a peak in late March, and subsided by early June, with a median duration of 21 weeks [5]. Despite a 92% increase in the number of tests performed during and after the pandemic, there was a dramatic 98% drop in hMPV circulation during the period of 2020–2021, likely due to widespread public health measures, which suppressed many respiratory viruses. The 2021–2022 season showed atypical and prolonged circulation (35 weeks) with regional variability, reflecting a period of viral re-emergence and ecological disruption which affected seasonality patterns during and after the pandemic [5]. This non-typical 35-week duration of hMPV activity in the United States was restored to the pre-pandemic seasonal model during the 2022–2023 and 2023–2024 periods. Prior to the COVID-19 pandemic, the circulation of hMPV typically succeeded that of RSV, whose seasonal outbreaks predominantly spanned from October through April. Both viruses commonly exhibited overlapping activity during the winter period [5]. Data from CDC, concerning six seasons prior to the pandemic, demonstrate that the median number of hMPV-positive tests was 16,096.5, with a peak of positive tests during the period of 2017–2018 (4.1% of the PCR tests performed). However, during 2020–2021, the initial COVID-19 period, there was a significant drop in hMPV-positive tests (0.07% of the PCR tests performed) representing the decrease in hMPV circulation during this timeframe (Figure 3).

**Figure 4:**
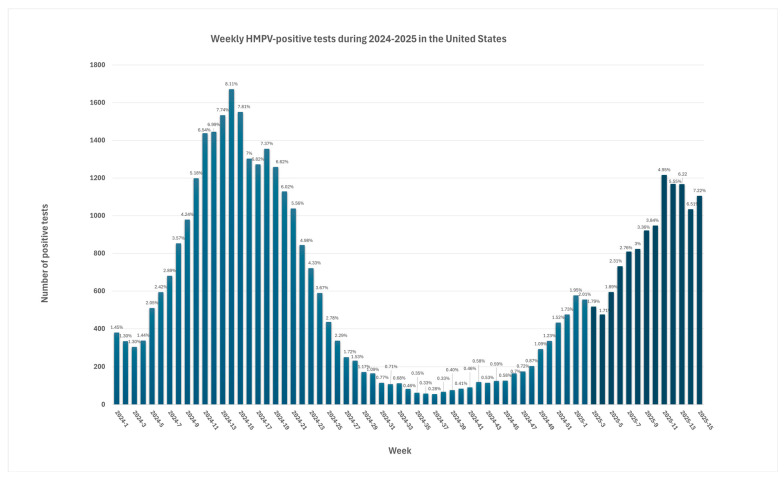
Weekly hMPV-positive tests during 2024–2025 in the United States. Labels on the chart represent positivity rates [50].

The percentage of positive hMPV during the following three seasons reached pre-pandemic levels [5]. During the first 15 weeks of 2025, the number of hMPV-positive test shows a steady increase, reaching a peak of 1216 positive tests during the middle of March of 2025. According to the data from The National Respiratory and Enteric Virus Surveillance System (NRVESS) dashboard, hMPV positivity was estimated at 7.22% during the 15nth week of 2025. This was the highest percentage of positive hMPV PCR tests of this year, possibly following typical seasonal patterns [50] (Figure 4).

hMPV can affect individuals across the entire age spectrum; however, it is most commonly encountered in children below the age of five years globally [6,8,10,12,49]. Elderly people and immunocompromised patients with hMPV are also susceptible to an acute respiratory illness. Recent data support an estimated hospitalization rate of 231 per 100,000 older adults, equating to about 122,000 cases in 2019 [7]. Although both hMPV and RSV affect pediatric populations, they differ in terms of high-risk groups and clinical management approaches. Hospitalized children with hMPV infection tend to be older compared to those admitted with RSV. In contrast, severe RSV-related illness primarily affects very young infants [6,55]. Bronchiolitis with a need for high-flow oxygen therapy is more commonly associated with RSV. In contrast, hMPV cases more frequently present with pneumonia and have a higher incidence of requiring mechanical ventilation [5]. Data from the CASCADIA Community-Based Cohort—Oregon and Washington for the years 2022–2024 demonstrate an overall incidence of clinically apparent hMPV infection of 7.5 per 100 (95% CI = 6.7–8.4) persons per year. The highest incidence was reported in preschool children aged 2–4 years measuring 19.5 (95% CI = 14.9–25.7) and reaching a peak of 41.3 (95% CI = 29.2–58.4) during January to March. Interestingly further analysis exhibited that hMPV infections tended to cluster within households and different households had different levels of infection risk highlighting the role of close interpersonal contact in the spread of the virus [6].

### 3.2. Argentina and Mexico

Between 2011 and 2013, a multicenter study in Argentina assessed severe lower respiratory tract infections (LRTIs) in preterm children under two years of age. Among 664 eligible cases, 8.1% tested positive for hMPV was identified in 2 of 60 near-fatal respiratory failure cases, with no associated deaths reported [52]. Interestingly, the study of Libster et al. reported that hMPV was generally associated with less severe illness in hospitalized children compared to other respiratory pathogens in the study. However, children admitted with hMPV-related LRTI, who were born to asthmatic mothers, exhibited an increased risk of life-threatening disease (LTD) [56]. These results could be in accordance with the fact that hMPV infection can elicit a complex immune response characterized by a predominant T-helper 2 (Th2) cytokine profile, including elevated IL-4, IL-5, and thymic stromal lymphopoietin, which resembles the immune patterns observed in asthma [57,58]. Additionally, hMPV may induce Th17-associated cytokines such as IL-1β, IL-6, and IL-17, contributing to airway inflammation [59]. A study conducted during the 2023–2024 winter season in Mexico investigated the viral etiology of acute respiratory infections in 390 hospitalized children under five years of age. hMPV was identified in 17.4% of cases, making it the third most prevalent virus detected. Among the fourteen cases transferred to the intensive care unit (ICU), hMPV was detected in 28.6%. Among the four reported fatalities, hMPV was co-detected in two cases alongside SARS-CoV-2, highlighting its potential contribution to severe disease outcomes in pediatric populations [53].

### 3.3. Europe

An analysis of hMPV surveillance data from 2012 to early 2025 revealed consistent seasonal patterns, with weekly positivity rates calculated as the proportion of confirmed hMPV cases among the tested samples. Excluding the COVID-19 pandemic seasons (2020/2021 and 2021/2022), hMPV typically exhibited winter seasonality, peaking between weeks 52 and 3 in 60% of seasons, and between weeks 12 and 17 in 40%. The highest recorded peak occurred in week 49 of the 2021/2022 season (10.33%) following the relaxation of pandemic-related restrictions. The median peak positivity was 5.54% (IQR: 5.07–6.09%). During the 2024/2025 season, the peak activity reached 4.6% in weeks 52 and 1, aligning with historical patterns and classified as moderate. Age-specific trends were also consistent, with children aged 0–4 years showing the highest positivity, peaking at 10.73% in week 52 of 2024—within the historical median for this group (11.11%, IQR: 10.34–12.04) [11]. A retrospective case series conducted at Scarborough General Hospital, UK, analyzed adult patients (≥18 years) with confirmed hMPV infection between 31 August 2022, and 1 September 2023. A total of 38 cases were identified, with the majority (76.3%) occurring in individuals aged over 65 years. Clinical presentation typically included influenza-like symptoms, and laboratory data included elevated C-reactive protein (CRP) and white blood cell count (mainly neutrophilia) and an absence of radiological abnormalities. Most patients were discharged, while five deaths occurred, all in patients over 65 years, 80% of whom had pre-existing comorbidities and concurrent acute conditions [60].

A multicenter study in North and Central Italy analyzed 11,577 respiratory samples collected between 1 December 2018, and 30 April 2019. hMPV was detected in 2% of tested samples (222/11,008), with positivity rates varying between 1.2% and 4%, independent of patient characteristics. hMPV circulation began increasing from week 9 of 2019, peaking in week 16 (12.2%). Pediatric patients accounted for 52.7% of hMPV-positive cases, while 30% were individuals over 65 years. Among hospitalized hMPV cases across the entire age spectrum, 11.3% required intensive care [61].

### 3.4. China and Other Asian Regions

The Pneumonia Etiology Research for Child Health (PERCH) study included children aged 1 to 59 months who were hospitalized with suspected severe pneumonia, along with age- and season-matched community controls, across seven countries in Africa and Asia. hMPV was more commonly detected in hospitalized cases compared to controls and in the control group, hMPV was more frequently detected within the group with respiratory tract illness. Children aged 6–11 months (8.8%) and 12–23 months (7.4%) had the highest rates of hMPV-positive cases, significantly higher than in the younger (6.1%) and older (5.2%) age groups (*p* < 0.03). Among the cases testing positive for hMPV, 65.2% were infants under one year of age [49].

### 3.5. China

China, which covers an extensive geographic area and heterogeneous climatic conditions, demonstrates significant regional differences in the incidence, seasonality, and molecular distribution of respiratory pathogens [62]. At the end of December 2024, a spike in ARTIs during the last weeks of 2024 was reported in northern regions of China following the seasonal detection of respiratory viruses including a significant increase in hMPV cases raising public concerns. Analysis of the *F-gene* from 28 hMPV strains reveals that the predominant genotype shifts depending on the surveillance year, with the most prevalent genotypes in China being A1, B1, and B2 [9]. Data from 2003 to 2023 report a prevalence of 0.97% to 15.88% across different regions, with the highest prevalence observed in Chongqing city, followed by Henan and Tianjin. hMPV distribution was significantly lower in individuals above the age of 5 years old. Higher prevalence occurred during spring and winter. Regarding seasonal distribution, individuals presenting with ARTIs during the spring season exhibited an approximately ninefold increased risk of hMPV infection compared to those sampled in the summer and autumn months. Summary data indicate the circulation of at least five distinct hMPV lineages (A1, A2b, A2c, B1, and B2) across China, with substantial regional variation in their distribution. Among these, the lineages A2b, B1, and B2 have emerged as the dominant strains nationally [62]. Evidence concerning ARTI patients in Henan between 2017 and 2023 reports a hMPV-positive rate of 6.71%, with children below the age of five presenting the highest detection rate at 7.78%. Seasonal outbreaks were reported mainly in spring during 2018 and 2019, with peak detection rates estimated at 31.11% in May 2018 and 19.57% in May 2019, followed by a remarkable peak of 42.11% in November 2020 that lasted until January 2021. After this period and until March 2023, the positive rates ranged between 0 and 8.7%. The predominant lineages identified were A2c111nt within subtype A and B2 within subtype B [63]. Epidemiological data from Shanghai during 2014–2023, on hospitalized patients with respiratory tract infection, exhibited similar results, with the highest hMPV-positive proportions being in the age group of 6–11 months (5.39%). The seasonality of hMPV spread in this area presented with a typical pattern during the pre-pandemic period. The outbreak seasons included winter and spring with peaks of hMPV-positivity seen from February to April during the period of 2014–2019. This seasonal model disappeared during the COVID-19 pandemic period of 2020–2022. The widespread implementation of non-pharmaceutical interventions (NPIs) significantly disrupted the typical seasonal circulation of hMPV leading to altered peak timing and patterns post-pandemic. The following years were characterized by a reversed seasonality pattern with hMPV outbreaks reported during summer and autumn in the area of Shanghai [10].

In Putian, a city with a distinct subtropical climate located in the southeast of China, hMPV presented a positivity of 9.1% during the 2023–2024 season and was among the most common pathogens participating in co-infections, mostly with Influenza B. Viral distribution exhibited notably higher detection frequencies in infants, toddlers, and preschool-age groups and maintained a consistently high positivity rate year-round, with a pronounced peak in incidence observed during the winter months [51]. Data from Beijing examining the prevalence of various pathogens pre- and post-pandemic support that in 2023, along with other viruses, the count of detected hMPV cases in hospitalized infants was the highest of the past six-year period. Post-pandemic data support a higher incidence of most viral agents including hMPV present during the fourth quarter [64]. In the Hangzhou region during 2022 and 2023, hMPV was among the most commonly detected viruses in children with bronchiolitis under the age of two years with a detection rate of 8.8%. During 2023, there was a shift in the seasonal distribution of hMPV compared to 2022, with a peak in detection observed during November and December. However, the pre-pandemic hMPV distribution mostly occurred during spring [65,66]. In Suzhou, during a period of four years from 2020 to 2024, hMPV was detected at 3.7% among hospitalized children with ARTIs. In this study, the peak hMPV distribution appeared between July to December 2022, after the release of the COVID-19 restriction policy. However, during 2023 and 2024, the monthly numbers of hMPV detected cases decreased by 73.2% and 30.3%, respectively. During the four-year period, the highest percentage of hMPV-positive tests was observed among children below the age of five years old [65]. In central China in the area of Jingzhou, hMPV was among the seven most common pathogens in children below the age of 18 with ARΤIs and remained the third most common pathogen in this patient group during 2022 and 2023. The rate of positive cases reached a peak in April and November 2022 as well as October 2023. hMPV presented as a single as well as a co-infection with Influenza B, Human Rhinovirus, Mycoplasma, RSV, and Adenovirus [67]. According to pre-pandemic data from a 10-year study (2010–2019) in southern China, hMPV was identified in 3.5% of pediatric ARTI cases, predominantly among children under six years of age. Its prevalence peaked primarily during the spring season. RSV was the most frequent co-detected pathogen (5.3%). Phylogenetic analysis showed that the sequenced hMPV strains were distributed across the following sublineages, A2b (1.9%), A2c (31.5%), B1 (50.0%), and B2 (16.7%), with the leading subtype being B1 [66].

### 3.6. hMPV Trends in Other Asian Countries

Data from a nationwide surveillance study in Korea between 2017 and 2023, concerning individuals under 20 years of age, showed a pre-pandemic detection rate of hMPV virus no less than 9.7%. However, there was a zero detection rate during the pandemic period of 2020–2021. hMPV re-emerged, exhibiting atypical seasonality and unusually elevated detection rates. By 2023, the hMPV seasonal distribution had nearly returned to pre-pandemic patterns, though the peak activity remained delayed by approximately 1–3 months. The mean pre-pandemic age of hMPV prevalence was 2.3 ± 2.0 years of age, while in 2023, the mean age was 3.6 ± 2.5 years of age, presenting a statistically significant increase [68]. The changes in hMPV seasonality and incidence during and after the COVID-19 pandemic were largely driven by widespread NPIs, which reduced virus transmission and healthcare utilization, leading to lower detection rates. After NPIs were lifted, decreased population-level immunity due to the interruption in virus circulation likely contributed to increased susceptibility and atypical, intensified outbreaks of respiratory viruses like hMPV [68].

A ten-year retrospective study in Taiwan, from 2013 to 2023, reported temporal fluctuations in the prevalence of hMPV, with a marked peak in 2021, and an absence of detected cases between 2018 and 2020, as well as in 2022. The A2 lineage was the predominant strain, followed by B2. hMPV infections were detected throughout most of the year in Taiwan, with the highest incidence observed between March and May, reaching a peak in April. Penghu County exhibited the highest detection rate, followed by Changhua County and Hsinchu County, while some regions reported no confirmed cases. The findings indicated that children aged 0 to 4 years accounted for the highest proportion of positive cases. Moreover, a statistically significant difference in virus prevalence was observed between individuals under 4 years of age and those over 60 (*p* < 0.001) [69].

The epidemiological trends of hMPV in children under ten years were analyzed in Tokyo, Japan, from January 2015 to June 2023. Prior to the COVID-19 pandemic (2015–2019), hMPV activity consistently peaked in spring (March–May) without overlapping with RSV seasons. Following the implementation of social restrictions in April–May 2020, hMPV circulation was undetectable until 2021. In 2022, an atypical seasonal shift occurred, with peak detection from May to November. Normal seasonality resumed in 2023, with a peak in May. Age distribution also shifted, with the most affected age group increasing from 1 year pre-pandemic to 3 years post-pandemic (2021–2023) [70]. A second study conducted in Tokyo between January 2021 and June 2024 reported hMPV positivity rates of 6.6% in children aged 0–9 years and 0.2% in adults over 20 years. Among adults aged 20–59 and those over 60, the hospitalization rates due to hMPV infection were 64.3% and 76.9%, respectively—the highest compared to rhinovirus/enterovirus (RV/EV), RSV, and parainfluenza virus type 3 (PIV3). In the older adult group, 2 fatalities were reported among 13 hospitalizations [71].

According to data from India, the pooled prevalence of hMPV infection among children with acute respiratory illness was estimated at 5% (95% confidence interval: 4–6%) during the time period of 2007–2019, whereas between 2020 and 2024 the prevalence was 4%. Subgroup analysis reported a higher prevalence in children younger than 5 years of age (6%) versus older counterparts (2%). Further analysis revealed highest though not significant prevalence of 7% in the Northeast followed by 6% in both the North and West, 5% in the South and 3% in the East [8]. In Gorakhpur, India, during the period between May 2022 to April 2023, the prevalence of hMPV positivity among children under 5 years of age with ARTI was 1.4%. The peak hMPV detection rates during this timeframe occurred in October (7.21%). Co-infection with other respiratory viruses such as Influenza A and Human Adenovirus was also detected. Eastern Uttar Pradesh, India, experiences a predominantly humid subtropical climate with dry winters, which may significantly influence the transmission dynamics of various respiratory viruses. hMPV cases typically begin to appear in September, peak in October, and gradually decline, with the lowest incidence observed during November and December [54].

The Bangladesh cohort of the PERCH study (2011–2014) reported hMPV detection rates of 14.6% in children with severe pneumonia and 20.9% in community controls. Among pneumonia cases, the highest prevalence was observed in children aged 6–11 months, while in community controls it was highest in those aged 12–23 months. Seasonal peaks occurred during August–November 2012, January–March 2013, and September–November 2014 [49]. Between 2014 and 2018, hMPV was the third most frequently detected viral pathogen in children under 2 years of age with acute lower respiratory infection, with a detection rate of 11.7%, following RV/EV and RSV. hMPV activity predominantly occurred from December to March, subsequent to the RSV seasonal peak, with the highest detection rates observed in children aged 6–11 months and 12–17 months [72]. In a comparative study in severely malnourished Bangladeshi children, involving 360 pneumonia cases and 334 controls, hMPV was identified in 4.5% of cases and 1.5% of controls. hMPV, along with RSV, influenza virus, and HPIV, showed a statistically significant independent association with pneumonia, with an odds ratio of 2.7 (95% CI: 1.3–5.5), indicating a notable link between hMPV infection and the occurrence of pneumonia in pediatric patients [73].

A study conducted in Iran between January 2016 and March 2017 on hospitalized children under 14 years with ARTI reported an hMPV detection rate of 2.64%, with the highest prevalence in children under three years of age. The most frequent hMPV co-infections identified were with adenovirus and influenza A virus [74]. In Pakistan, hMPV was present in 6.1% of children with pneumonia. The highest prevalence was observed in the age group of 1–5 years. The annual hMPV distribution in 2015 and 2016 was 5.3% and 7.7%, respectively [75].

### 3.7. Australia

In the western Australian region between 2010 and 2021, there were 43,322 hMPV tests in children <5 years with respiratory infection. The hMPV testing rate for infants <12 months were 248 per 1000 child-years while the testing rates in all age groups increased during this timeframe. The positivity rate in children was 4.09% in infants compared to 5.73% in toddlers of 1–5 years of age [76].

## 4. Transmission and Clinical Manifestations

The modes of transmission and epidemiological features of hMPV closely resemble those of other respiratory viruses such as RSV and influenza. hMPV exhibits a seasonal pattern of circulation, predominantly occurring in late winter and spring in temperate regions, though cases have also been reported during summer and early autumn [5]. The virus is primarily transmitted through respiratory droplets and direct or indirect contact with contaminated secretions, including saliva and aerosols. Transmission may occur via large droplets over short distances or smaller airborne particles capable of remaining suspended for longer periods. Following an incubation period of approximately 3 to 5 days, the virus rapidly infects the respiratory tract after inoculation of the nasopharyngeal mucosa [77]. Notably, both symptomatic and asymptomatic individuals can facilitate transmission, with evidence suggesting a potential for presymptomatic spread underscoring the challenges in early detection and containment [12].

Since its identification in 2001, hMPV has been recognized as a significant causative agent in respiratory tract infections across the entire age spectrum. The clinical manifestations of hMPV infection encompass a broad spectrum, ranging from mild upper respiratory tract infection (URTI) including fever, rhinorrhea, pharyngitis, conjunctivitis, and cough to more severe LRTI, including fever, tachypnea, cough, hypoxia, wheezing, croup, and pneumonia [12,13]. Radiographic evaluation may reveal characteristic findings such as lung hyperinflation, pulmonary infiltrates, and peribronchial thickening. Clinically, the symptoms of hMPV infection often overlap with those observed in RSV infections, including cough, fever, dyspnea, rhinorrhea, shortness of breath, rashes, and hypoxia [4,12].

Similar to other respiratory viruses, hMPV has been identified in pediatric patients presenting with acute otitis media co-existing with either URTI or LRTI [78,79]. The post-viral inflammatory response can compromise Eustachian tube function, leading to obstruction and creating favorable conditions for secondary bacterial infection of the middle ear [13]. Additionally, the detection of hMPV in the nasal secretions of 6% of children diagnosed with acute otitis media supports its potential role as a contributing etiological agent alongside other common pathogens [78].

hMPV not only affects infants and children but is also known to cause acute respiratory illness in immunocompromised individuals, the elderly, and patients with comorbidities such as asthma or malignancies [7]. The involvement of hMPV in the exacerbation of asthma has been reported in multiple studies [80,81]. In adult populations, hMPV has been identified in cases of acute asthma exacerbation requiring hospitalization. Beyond asthma, the virus has also been implicated in various other respiratory illnesses in adults, including pneumonia, influenza-like illness, and chronic obstructive pulmonary disease (COPD) [82]. Although the overall incidence of hMPV infection is generally lower in adults compared to children, its ability to cause disease across all age groups remains a significant clinical consideration.

hMPV is a respiratory pathogen capable of causing a wide spectrum of complications, particularly affecting the respiratory system. Although commonly resulting in mild illness, hMPV has been linked to severe pneumonia in healthy adults and life-threatening acute respiratory distress syndrome (ARDS), often requiring intensive interventions such as extracorporeal membrane oxygenation (ECMO) [83,84]. In infants, hMPV can lead to bronchiolitis obliterans and significant respiratory failure necessitating prolonged ventilation [85]. A considerable number of pediatric patients require pediatric ICU admission and various levels of respiratory support [86]. Co-infection with SARS-CoV-2 has been associated with fatal outcomes in children, underscoring the dangers of concurrent viral infections [87]. Cardiovascular complications include myocarditis and cardiogenic shock in both men and women [88], while neurological manifestations range from seizures and encephalopathy to long-term cognitive and motor impacts [89,90,91]. Rare complications include acute hemorrhagic edema of infancy and rhabdomyolysis with multiorgan dysfunction [92]. These diverse and sometimes fatal outcomes highlight the need for heightened clinical awareness and supportive care strategies in managing hMPV infections [93]. These complications are summarized in Table 1.

## 5. Diagnostic Methods

Over the past two decades, a variety of molecular techniques have been developed and refined for the detection of hMPV, each with its own advantages and limitations. Conventional reverse transcription–polymerase chain reaction (RT-PCR) methods, initially widely used, target conserved regions like the *F* and *N genes* of hMPV for detection and genotyping [94,95]. While these methods provided a foundation for molecular diagnosis, they have gradually been replaced by more sensitive technologies, such as reverse transcription–quantitative polymerase chain reaction (RT-qPCR). This method is now considered the gold standard due to its higher sensitivity, lower contamination risk, and ability to differentiate hMPV subgroups. Multiplex RT-qPCR assays further enhanced diagnostic capabilities by allowing the simultaneous detection of hMPV and other respiratory pathogens [96].

Emerging isothermal amplification methods like loop-mediated isothermal amplification (LAMP) and recombinase-aided amplification (RAA) have introduced simpler, faster alternatives that operate without complex thermal cycling equipment. These methods are especially valuable in low-resource or point-of-care settings [62,97,98]. Innovations such as clustered regularly interspaced short palindromic repeat (CRISPR) and CRISPR-associated proteins (Cas) (CRISPR-Cas12a) combined with isothermal amplification have further expanded the diagnostic options by offering visual detection capabilities with high specificity and speed [99,100]. Advanced techniques like metagenomic next-generation sequencing (mNGS) have enabled the identification of novel or uncommon pathogens, offering whole-genome resolution and deep insights into viral dynamics [101,102]. Despite their significant investigational and identification value, these techniques remain under evaluation for routine clinical use due to factors such as high cost, complexity, and limited accessibility. Virus isolation, although still considered a gold standard for vaccine development and pathogenesis studies is hindered by hMPV’s slow replication and low cytopathic effect (CPE), making it less practical for routine diagnosis [103,104]. The methods are summarized in Table 2.

Advancements in molecular diagnostic techniques have significantly improved the detection and characterization of hMPV, with RT-qPCR emerging as the gold standard [95]. Isothermal amplification methods such as LAMP and RAA, along with CRISPR-based assays, offer rapid, equipment-free alternatives suitable for point-of-care use [97,98,99], while mNGS provides comprehensive genomic insights, facilitating the identification of novel or uncommon strains [100,101]. These innovations hold the potential to improve diagnostic accuracy, enable timely clinical decision-making including the avoidance of unnecessary antibiotic use, and enhance surveillance efforts, particularly in diverse healthcare settings.

## 6. hMPV and Comorbidities

hMPV has emerged as a significant respiratory pathogen, with its impact on outcomes in individuals with pre-existing comorbidities being under considerable research.

Cystic fibrosis (CF) mortality is largely driven by respiratory complications caused by chronic bacterial infections that lead to airway damage and disease progression, yet the role of viral respiratory infections remains less clear [105]. In a cohort of children with CF, a notably high incidence of hMPV infection was observed, with infection rates of 47.8% and 47.3% among those aged 7–12 and 13–18 years, respectively. hMPV was associated with increased LRTI events in CF children during infection periods; however, these increases, including hospitalization rates, were not statistically significant [106]. In an Australian cohort, the prevalence of hMPV was comparable between individuals with and without CF (2.4% and 3.4%, respectively), with a significantly higher detection rate in pediatric CF patients compared to adults (*p* = 0.0165). Both hMPV and RSV were more frequently identified in children, irrespective of CF status, aligning with their established role as common etiological agents of viral respiratory infections in pediatric populations. Notably, hMPV was undetected in adult CF patients, and RSV was identified in fewer than 1% of this group [105].

Pediatric oncology patients are particularly vulnerable to infections, primarily due to treatment-induced neutropenia. hMPV ranks among the ten most frequently identified respiratory viruses in this population, detected in 3.3–13.5% of acute respiratory infection cases and in 0.4–44% of febrile neutropenia episodes. hMPV is often the sole pathogen, though co-infections with other viruses or bacteria are common. In some instances, hMPV has been implicated in severe outcomes, including fatal cases, particularly among hematopoietic stem cell transplant recipients [107]. Notably, hMPV was responsible for a documented outbreak in a pediatric oncology unit [108]. A retrospective study of 30 oncology pediatric patients positive for hMPV revealed that 46.7% developed lower respiratory tract infections, while 53.3% had upper respiratory involvement. The median duration of illness was 6.5 days, with 66.6% requiring hospital admission. Notably, none of the patients needed mechanical ventilation, although one case required prolonged inhaled steroid and bronchodilator therapy. All children recovered fully, indicating that despite hMPV infection being able to cause considerable disease in this population it is not typically fatal [109].

The incidence of hMPV in immunocompromised patients, including those with hematological malignancies, can be as high as 40%, with a slightly higher rate observed in adults compared to children [110]. hMPV can cause both upper and lower respiratory tract infections, with symptoms ranging from mild nasal congestion and cough to severe pneumonia, hypotension, and shock if LRTI develops. Risk factors for LRTI include steroid use, low lymphocyte count, and early onset of infection after hematopoietic cell transplantation (HCT) [110,111]. Mortality associated with hMPV infection ranges, depending on the severity of the illness [110]. hMPV was also identified in immunocompromised patients with acute myeloid leukemia (AML) who developed severe respiratory symptoms. Despite treatment with ribavirin, outcomes varied: one patient improved, while others experienced rapid deterioration and death. These cases suggest hMPV as a significant contributor to respiratory complications in neutropenic AML patients, with outcomes potentially worsened by their immunosuppressed state [112].

## 7. hMPV in the Perinatal Period

Pregnant women represent a distinct population due to the immunomodulatory effects of gestation. Thus, they are at an increased risk for complications from seasonal and pandemic viral infections including hMPV [113,114]. hMPV infection has been accused of causing acute respiratory syndrome in pregnant women [114,115]. Furthermore, a prospective study that included asthmatic and non-asthmatic pregnant women reported that in women with asthma, laboratory-confirmed viral infections (including hMPV) were significantly associated with adverse maternal health outcomes, with 60% of infections correlating with episodes of uncontrolled asthma and an elevated risk of complications [116]. In this study, women with asthma who tested positive for respiratory viruses by PCR (including hMPV) had significantly higher odds of developing preeclampsia or pregnancy-induced hypertension, even after accounting for the established risk factors for preeclampsia [116].

There are a few case reports on hMPV infections during pregnancy, none of which report vertical hMPV transmission. A 24-year-old pregnant woman at 30 weeks’ gestation developed respiratory insufficiency following the onset of fever and urinary tract infection. Despite normal chest X-rays, she progressed to significant hypoxia with bilateral alveolar consolidations identified on a CT scan and tested positive for hMPV. At 36 weeks of gestation, she gave uncomplicated birth to a healthy female neonate [31]. A 40-year-old pregnant woman at 29 weeks’ gestation with a history of asthma was admitted to the ICU due to worsening respiratory symptoms caused by hMPV infection. After receiving ICU care, her condition gradually improved and she was discharged. At nearly 39 weeks of gestation, she underwent uncomplicated vaginal delivery of a 3600 g female neonate, with no clinical evidence of infection in the newborn [117]. A 36-year-old woman at 31 weeks of gestation was admitted for respiratory symptoms secondary to an hMPV infection. Her clinical course was complicated by a secondary lower respiratory tract infection requiring antibiotic therapy. She showed gradual improvement and was discharged on hospital day 8. At 39 weeks of gestation, she underwent an elective repeat cesarean delivery of a healthy 3230 g female neonate [117]. An 18-year-old, late-preterm pregnant woman developed acute respiratory distress syndrome after hMPV infection and required ICU admission, intubation, and primary cesarean delivery due to worsening respiratory function. Her condition further deteriorated postpartum, necessitating extracorporeal membrane oxygenation. She eventually recovered with supportive care. At birth, the neonate required resuscitation; however, by the second minute of life, the infant had been stabilized and showed no signs of infection [114]. A 34-year-old term pregnant woman developed pneumonia due to hMPV shortly after cesarean delivery, presenting with worsening cough, bilateral wheezing, and pleural effusion with left basal consolidation. She was transferred to the ICU for anticipated respiratory decompensation. She showed progressive clinical improvement, leading to discharge on day 6 with stable oxygen saturation on room air [118]. A 37-year-old woman at 7 months of gestation was admitted to the ICU with severe respiratory symptoms and was diagnosed with hMPV pneumonia, confirmed by PCR and supported by CT findings of bilateral ground-glass opacities. Following supportive care and antimicrobial therapy, her respiratory status improved, allowing for de-escalation of oxygen support and discharge in stable condition after six days [118].

Studies have examined the possible effects of viral respiratory infections on both the mother and the fetus, linking them to adverse birth outcomes in the general frame of a systemic inflammatory response [119]. The intrauterine environment may exert a substantial influence on the developing immune system, triggering both innate and adaptive immune responses, potentially mediated by placental inflammation [119]. Maternal hMPV infection has not been found to undergo intrauterine transmission; however, it has been linked to altered birth outcomes [113,120]. The study of Lenahan et al. on the effects of hMPV and other viral infections on birth outcomes in Nepal correlated hMPV febrile illness during pregnancy with an increased risk of SGA in infants (risk ratio 1.7; *p* = 0.031). In the same study, the median maternal symptom duration was five days. Nearly half (43.6%) of pregnant women infected with hMPV also experienced a viral co-infection, primarily with rhinovirus, and to a lesser extent, coronavirus and parainfluenza [113]. Murphy et al. reported a significantly lower birth weight and length in neonates born from non-asthmatic mothers with at least one PCR-confirmed common cold, including hMPV, during pregnancy [116].

Data from South Africa revealed an increased susceptibility of LRTI hospitalization in hMPV-infected infants under 6 months of age that have been exposed to but are uninfected by maternal HIV infection (HEU), compared to a group of unexposed and uninfected age-matched infants born from HIV-infected mothers [112]. Studies aiming to further investigate this phenomenon found a decrease in hMPV-neutralizing antibody levels in HEU infants, compared to matched HIV-1 unexposed controls. Concurrently, the transfer of hMPV-neutralizing antibodies across the placenta was significantly reduced in women with HIV-1 compared to HIV-negative counterparts. However, lower cord blood titers of hMPV-neutralizing antibodies were not significantly correlated with early hMPV infection in infants under six months of age in this study [121]. The impact of intrauterine programming on the offsprings’ immune system against viral infections such as hMPV has also been examined in animal models. Female adult mice gestated in a hyperthyroxinemic intrauterine environment experienced a more severe hMPV infection, consistent with the increased histopathological pulmonary inflammation after seven days of infection. These results support the notion that gestation under hypothyroxinemia stimulated an extrauterine altered inflammatory response against hMPV infection [122].

## 8. Treatment and Prevention Strategies—Recent Data

### 8.1. Putative Antiviral Agents

Currently, the therapeutic options for hMPV infections are predominantly supportive. Symptomatic management includes the administration of antipyretics, such as acetaminophen or ibuprofen, to control fever. Intravenous fluids may be required in cases of dehydration, particularly when oral intake is insufficient [16]. In severe presentations involving respiratory failure, patients—especially those with underlying cardiopulmonary conditions or compromised immune systems—may require supplemental oxygen or mechanical ventilation. While most individuals recover, infection control measures such as droplet precautions are essential to prevent transmission [15,16].

Ribavirin, a nucleoside analog commonly used in the treatment of hepatitis C and other viral infections, is the most frequently administered antiviral agent. However, due to its potential teratogenicity and adverse effects such as hemolytic anemia, its use is generally limited to severe hMPV cases [12]. Recent research on hMPV has identified several promising therapeutic candidates targeting various stages of the viral lifecycle. Computational studies have highlighted the potential of the N protein as an antiviral target, with compounds such as ZINC85629735 (M1) and ZINC85569125 (M3) showing strong binding affinities and favorable stability in molecular dynamics simulations, making them promising drug candidates [123]. Other interventions, such as the use of interferons (IFN-ε and IFN-λ), have been shown to modulate immune responses and reduce viral loads without inducing inflammation, suggesting their therapeutic potential [124,125]. Moreover, FDA-approved drugs like Probenecid have been repurposed for hMPV treatment, exhibiting broad-spectrum antiviral effects [126]. The non-nucleoside inhibitor JNJ-8003 has also shown promise, inhibiting viral replication by targeting the RNA-dependent RNA polymerase complex [127].

Natural glycans such as lacto-N-neotetraose (LNnT) and glycosaminoglycans—especially heparin—have demonstrated strong, dose-dependent inhibition of viral binding to the host cell via interactions with the F protein, highlighting their potential as viral entry inhibitors [30]. Additionally, natural compounds, including Ginkgolic acid (GA), Fumarprotocetraric acid (FUM), and Geraniin (GE), have demonstrated effective antiviral activity, primarily by inhibiting viral entry and replication in the early stages of infection [128,129]. Quercetin, a bioactive flavonoid, exhibited multi-target antiviral effects by interacting with the viral matrix protein and reducing oxidative stress and inflammation, with nanoformulations overcoming its solubility and bioavailability limitations [130]. Researchers identified monoclonal antibodies (mAb 5-1 and mAb 338), with mAb 5-1 demonstrating broad neutralizing activity against both RSV and hMPV by targeting a unique epitope on the viral fusion protein, while mAb 338 effectively reduced viral replication and lung inflammation in hMPV-infected mice, showing both short- and long-term protective effects, thus highlighting their potential therapeutic applications [131,132]. These findings underscore the diverse approaches under investigation for hMPV treatment, from immune modulation to small molecule inhibitors, which hold significant potential for managing this respiratory pathogen. The summarized data is presented in Table 3.

### 8.2. Research on Immunization Strategies

Although there are currently no licensed vaccines or antiviral treatments for hMPV, several vaccine candidates are undergoing early-stage clinical evaluation. Neutralizing monoclonal antibodies (nMAbs) represent a fundamental component of vaccine-induced immunity and serve as critical therapeutic agents against various infectious agents [133]. Recent advancements in the structural biology of the hMPV F protein, along with prior insights from RSV and other enveloped viruses, have significantly propelled the development of nMAbs targeting hMPV [133]. Multiple strategies are under investigation for the development of effective vaccines against hMPV and related respiratory viruses, including live attenuated, virus-like particle (VLP), mRNA-based, and subunit vaccine platforms. A live attenuated rhMPV vaccine showed limited infectivity and immunogenicity in children leading to trial discontinuation [134]. In contrast, the VLP-based IVX-A12 demonstrated robust and sustained immune responses in older adults [135], while the bivalent Metavac^®^-RSV LAV induced strong mucosal and systemic immunity in preclinical models [136]. Stabilized prefusion F protein trimers and Gag-fused VLPs elicited potent neutralizing antibody responses, highlighting the role of structure-guided design [137]. Plant-derived adjuvants such as cpcQS-21 showed comparable efficacy to conventional adjuvants, offering a scalable alternative [138]. Additionally, the mRNA-1653 vaccine showed durable humoral responses against hMPV, and a novel dual-cleaved Pre-F protein design enhanced immunogenicity and structural stability [139]. Collectively, these studies underscore the promise of diverse, next-generation platforms in advancing hMPV vaccine development. Current research on the design of hMPV vaccines is summarized in Table 4.

Encouraged by the success of novel hRSV vaccines in phase III trials, current hMPV vaccine research emphasizes the structural characterization of viral antigens and their corresponding neutralizing epitopes. A growing repertoire of high-affinity nMAbs against hMPV has been discovered, some demonstrating the cross-neutralization of hRSV. These findings offer valuable perspectives for the development of both prophylactic and therapeutic interventions targeting hMPV and hRSV infections [140]. Such challenges have prompted a shift toward next-generation vaccine strategies, particularly those that are structure-guided and rely on the identification of potent neutralizing antibodies. Recent breakthroughs in vaccine technology, particularly those applied to hRSV, have demonstrated robust immunogenicity and favorable safety profiles in late-stage clinical trials [140,141]. These approaches, grounded in the precision of structural vaccinology and informed by epitope-specific antibody responses, now upgrade hMPV vaccine development.

Despite significant advances in hMPV vaccine development, several challenges remain. Antigenic variation in the hMPV genome, which alters protective epitopes, can undermine vaccine efficacy by failing to provide sufficient immunity against circulating strains [142]. Additionally, while live attenuated vaccines offer effectiveness, they pose safety risks, particularly for immunocompromised individuals, and inactivated vaccines may not generate robust immune responses [134]. Long-lasting immunity is essential for herd immunity, necessitating innovative strategies to extend the duration of vaccine-induced protection. The exploration of novel vaccine platforms, such as mRNA vaccines and nanoparticle-based delivery systems, could improve both safety and efficacy. Collaborative efforts among researchers and manufacturers are crucial to developing univalent or multivalent vaccines that target hMPV alongside other respiratory pathogens.

### 8.3. Challenges in Outbreak Control in Institutional Settings

Controlling infectious disease outbreaks—and hMPV transmission in particular—in institutional settings such as long-term care facilities (LTCFs) and pediatric long-term care facilities (pLTCFs) presents persistent and multifaceted challenges. A key factor contributing to outbreaks is the failure to consistently implement and adhere to fundamental infection-prevention protocols [143,144]. Despite recommendations from health authorities like the CDC, work restrictions for infected or exposed healthcare personnel are not always enforced, even though staff are frequently implicated in the transmission of viral infections such as hMPV. The presence of symptomatic employees during outbreaks highlights the critical need for the stricter enforcement of exclusion policies and underscores the importance of maintaining rigorous daily infection control practices [143,144].

Additionally, resource limitations in LTCFs may impede the execution of non-pharmaceutical interventions and other infection control strategies. In pediatric settings, the challenges are compounded by unique environmental and clinical factors, including the frequent use of medical devices, developmental behaviors that increase exposure risk, and the presence of communal activities such as schooling [145]. While targeted interventions—such as the employment of specialized infection control personnel and enhanced hygiene protocols—have shown promise in reducing outbreak incidence, the complete prevention of ARIs remains unlikely. Thus, strategies should prioritize minimizing pathogen transmission to prevent hospital transfers and maintain the continuity of essential services. These complexities emphasize the need for ongoing research and policy development to enhance outbreak preparedness and response in institutional care environments [145].

Outbreak control in childcare institutions is particularly complex due to the close contact among young children, who are highly susceptible to and efficient at transmitting infectious agents. Newly enrolled children are at a heightened risk of respiratory infections such as hMPV infection, which can subsequently affect caregivers, families, and the broader community [146,147]. Effective prevention relies on strict adherence to infection control measures such as hand hygiene, immunization compliance, environmental sanitation, and structured exclusion policies. However, asymptomatic shedding often precedes clinical illness, making proactive strategies and continuous education for staff and parents essential. Collaborative efforts with public health authorities and adherence to evidence-based guidelines are crucial for mitigating hMPV transmission and managing outbreaks in these high-risk environments [146].

## 9. Conclusions

hMPV remains a significant yet underrecognized contributor to acute lower respiratory tract infections, posing a substantial global health burden, especially among vulnerable populations such as young children, older adults, and immunocompromised individuals. Although advancements in diagnostic techniques—including RT-qPCR, mNGS, and immunoassays—have improved detection, clinical management continues to rely primarily on supportive care due to the absence of an approved vaccine or specific antiviral treatment. The virus, although only formally identified in recent decades, likely circulated for years beforehand, and its resurgence underscores the need for continued vigilance and research.

The data presented in this review provide a critical foundation for informing clinical and public health strategies aimed at mitigating the global burden of hMPV. Clinical risk stratification can be assessed by identifying the populations most vulnerable to severe hMPV infection, such as infants, older adults, and immunocompromised individuals. Insights into seasonal and geographic patterns of circulation can support the timing and allocation of diagnostic resources, while the characterization of cytokine responses may guide the development of targeted immunomodulatory therapies. Furthermore, the review’s synthesis of diagnostic advancements can aid clinicians and laboratories in selecting the most appropriate and sensitive detection methods to improve early diagnosis and patient management.

Current surveillance systems for viral respiratory infections, including hMPV, exhibit notable limitations in both timeliness and the integration of critical data streams. The lack of standardized, digitalized, and interoperable frameworks across EU/EEA countries hinders effective response coordination, particularly for emerging or underrecognized pathogens like hMPV. Many national systems still rely on outdated paper-based reporting, contributing to delays and fragmentation in data flow. Real-time diagnostics and the incorporation of electronic health records, mobile health applications, and other digital sources hold considerable promise in bridging these gaps by enabling earlier detection, better risk assessment, and more agile public health interventions [148,149,150]. Expanding molecular and genomic surveillance for hMPV, integrated with epidemiological and environmental data, is essential to improve outbreak prediction and response. Moreover, the COVID-19 pandemic has underscored the urgent need to modernize epidemic intelligence by leveraging big data and AI, ensuring that surveillance outputs are timely, comparable, and actionable at both the national and supranational levels.

Looking ahead, prioritizing the development of effective vaccines, particularly mRNA-based and multivalent platforms, is essential to mitigate the impact of hMPV across diverse population groups. Parallel efforts must focus on the discovery and evaluation of targeted therapeutics, such as monoclonal antibodies and novel small molecules, to expand treatment options. Strengthening surveillance systems, enhancing public awareness, and improving the sensitivity and accessibility of diagnostic tools—especially in resource-limited settings—are crucial to achieving timely intervention and control. Furthermore, large-scale epidemiological and immunological studies are needed to better understand hMPV’s seasonal trends, co-infection dynamics, and geographic distribution. Expanding clinical trials to include high-risk cohorts will be vital to ensure equitable access to emerging therapeutics and vaccines, ultimately reducing the morbidity and mortality associated with this re-emerging respiratory pathogen.

## Figures and Tables

**Table 1:** Severe clinical manifestations of hMPV infection.

Condition	Patient Description	Findings	Outcome	References
Severe Pneumonia	68-year-old man	Bilateral ground-glass opacities, hMPV confirmed via RT-PCR	Recovered with supportive care	[83]
ARDS	45-year-old man	Severe ARDS, required ECMO, hMPV confirmed	Recovered and discharged	[84]
Bronchiolitis Obliterans	3-month-old girl	Respiratory failure, CT: hyperinflation, atelectasis, hMPV confirmed	Discharged after 76 days with tracheostomy	[85]
PICU Admission	Pediatric population	18% PICU admission, 69% needed respiratory support	More severe in those with chronic conditions	[86]
hMPV + COVID-19	3 pediatric cases	Co-infection, ARDS, ground-glass opacities	All cases fatal despite ICU care	[87]
Myocarditis	68-year-old man	Heart failure, atrial flutter, hMPV, MRI: myocarditis	Discharged stable after 12 days	[88]
	58-year-old woman	Stress-induced cardiomyopathy, hMPV confirmed	Recovered, discharged after 9 days	
CNS—Encephalopathy	16 children	Various encephalopathy subtypes, hMPV confirmed	11 good outcomes, 5 poor outcomes	[89]
CNS—Seizures	4-month-old infant	Seizures, respiratory failure, hMPV detected	Recovered, seizure-free for 6 months	[90]
CNS—Status Epilepticus	15-month-old girl	Status epilepticus, pneumonia, pneumothorax, hMPV	Recovered without lasting effects	[90]
	18-month-old girl	Seizures, ARDS, atelectasis, hMPV confirmed	Recovered fully	
CNS—Multiple Cerebral Hemorrhages and White Matter Lesions	6-year-old girl (trisomy 13)	hMPV pneumonia, ARDS, DIC, MRI: hemorrhagic infarctions and signs of a demyelinating white matter lesion	Recovered with neurological deficits	[91]
Acute Hemorrhagic Edema of Infancy	8-year-old boy	Rash, edema, elevated CRP, splenomegaly, hMPV	Improved with NSAIDs and antihistamines	[92]
Rhabdomyolysis	4-year-old girl	MODS, ARDS, myoglobinuria, hMPV detected	Died despite intensive care	[93]

hMPV: human metapneumovirus; ARDS: acute respiratory distress syndrome; ECMO: extracorporeal membrane oxygenation; PICU: pediatric intensive care unit; CNS: central nervous system; MRI: magnetic resonance imaging; DIC: disseminated intravascular coagulation; MODS: multiorgan dysfunction syndrome; NSAIDs: nonsteroidal anti-inflammatory drugs; CRP: C-reactive protein.

**Table 2:** hMPV detection methods based on PCR and related technologies.

**PCR-based techniques used for molecular detection**					
RT-PCR	Conventional reverse transcription PCR widely used in early hMPV detection.	*F* and *N genes*	~1000 copies/reaction (GeXP multiplex assay)	**Less sensitive than RT-qPCR; declining use in clinical diagnostics; some commercial panels still include it.** **Process time: 3–5 h.**	**[94,95]**
RT-qPCR	Real-time quantitative RT-PCR; gold standard for viral RNA detection.	Various including *F* and *N genes*	10–100 copies/reaction	High sensitivity and specificity; allows for multiplexing; widely used in clinical labs. Process time: 1–3 h.	[96]
Nucleic acid isothermal-based amplification techniques					
LAMP	Isothermal amplification technique.	*M* and *N genes*	<10 copies/reaction	Fast, simple, does not require thermocycler; visual detection possible; higher sensitivity than RT-PCR. Process time: ~1.5 h.	[62,97]
RAA	Isothermal method with rapid results and minimal equipment.	*N gene*	100 copies/reaction	Rapid (15 min @ 39 °C); more efficient than some RT-qPCR methods in field settings.	[98]
Gene-editing and molecular detection methods					
CRISPR-Cas12a (with RT-RPA)	Gene-editing-based detection combined with RT-RPA and lateral flow.	*N gene*	<700 copies/mL	Results visible to the naked eye; ~30 min total time; 96.4% match with RT-qPCR results. Suitable for point-of-care testing.	[100,101]
mNGS	High-throughput sequencing for pathogen discovery and genomic analysis.	Whole genome	Not explicitly quantified; 80% detection in clinical samples	Detects novel/unknown viruses; high accuracy but lower sensitivity than RT-qPCR; useful in outbreak and research settings. Process time 5–10 days.	[102,103]
Classical virological methods					
Virus Isolation	Traditional method using cell lines (e.g., LLC-MK2).	hMPV cultivation and isolation	Not determined	Gold standard for pathogenesis and vaccine research; slow growth and low CPE in hMPV; confirmed via serological/molecular methods. Process time: 3–4 days.	[104,105]

RT-PCR: reverse transcription–polymerase chain reaction; RT-qPCR: real-time quantitative reverse transcription polymerase chain reaction; LAMP: loop-mediated isothermal amplification; RAA: recombinase-aided amplification; CRISPR: clustered regularly interspaced short palindromic repeat; Cas: CRISPR-associated proteins; RPA: recombinase polymerase amplification; mNGS: metagenomic next-generation sequencing; CPE: cytopathic effect; LCC-MK2: Louisiana Lung Cancer, Monkey Kidney 2.

**Table 3:** Putative hMPV antiviral agents.

Molecule/Compound	Mechanism of Action	Key Findings	References
ZINC85629735 (M1)	Inhibits the N protein of hMPV, which protects viral RNA from degradation.	Strong binding affinity with a docking score of −9.6 kcal/mol. MD simulations indicated favorable stability with an MM-GBSA binding energy of −81.94 kcal/mol.	[124]
ZINC85569125 (M3)	Inhibits the N protein of hMPV.	Strong binding affinity with a docking score of −10.7 kcal/mol. MD simulations showed favorable stability with an MM-GBSA binding energy of −99.63 kcal/mol.	
Interferon Epsilon (IFN-ε)	Induces *ISGs* such as *ISG15*, reducing hMPV replication.	IFN-ε shows higher sensitivity to hMPV inhibition compared to RSV. It activates a strong antiviral response and reduces viral replication, with minimal involvement of TLR pathways.	[125]
Interferon Lambda (IFN-λ)	Upregulated during hMPV infection, suppresses viral replication and dissemination.	Reduces viral load without triggering inflammatory pathology, preserves ciliated epithelial cells, and regulates macrophage recruitment.	[126]
Probenecid	Inhibits OAT3, a host protein required for viral replication, blocking virus-related signaling pathways.	Significantly inhibits hMPV replication in vitro and reduces lung viral titers in vivo without notable lung pathology, suggesting a broad-spectrum antiviral action.	[127]
JNJ-8003	Targets the RdRp complex of hMPV, inhibiting early transcriptional events.	Potent inhibition of RSV and moderate inhibition of hMPV replication. Binds to the capping domain of the L protein, preventing de novo RNA synthesis.	[128]
Lacto-N-neotetraose (LNnT)	Inhibits early-stage viral attachment by binding to the F protein.	Inhibits hMPV binding with 98% inhibition at 10 mM, IC_50_ = 1.88 mM. Shows promise as a glycan-based antiviral therapy.	[30]
Heparin	Inhibits early-stage viral attachment by targeting the F protein.	More potent than other glycosaminoglycans, with an IC_50_ approximately 30-fold lower than heparan sulfate.	
Ginkgolic Acid (GA)	Inhibits viral entry at the early stages of the viral lifecycle.	Reduces GFP-positive cells in A549 and Vero E6 cells, with IC_50_ values of 0.44 µM and 0.78 µM, respectively. Primarily targets viral entry mechanisms.	[129]
Fumarprotocetraric Acid (FUM)	Suppresses hMPV replication and exhibits anti-inflammatory effects.	Inhibits viral replication in LLC-MK2 cells, while also reducing viral RNA levels and mitigating viral cytopathic effects.	[130]
Geraniin (GE)	Suppresses hMPV replication and exhibits anti-inflammatory effects.	Inhibits hMPV replication in LLC-MK2 cells, reduces viral RNA levels, and protects mitochondrial function.	
Quercetin	Disrupts viral assembly, reduces oxidative stress, and modulates inflammatory pathways.	Attenuates hMPV-induced oxidative stress and inflammation in cell culture and animal models. Nanoparticles enhance stability and cellular uptake, showing promise in reducing viral load.	[131]
mAb 338	Targets the F protein of hMPV to prevent viral entry into host cells	mAb 338 significantly reduced viral replication, lung inflammation, and airway obstruction in hMPV-infected mice. The higher dose also lessened long-term lung hyperresponsiveness, indicating both short- and long-term protective effects.	[132]
mAb 5-1	Broad cross-neutralization of RSV and hMPV by binding to a unique epitope on the F protein, preventing viral fusion.	Demonstrated cross-neutralization of RSV and hMPV. Strong prophylactic efficacy in mouse models. Supports dual-virus therapeutic potential.	[133]

hMPV: human metapneumovirus RNA: ribonucleic acid; MD: molecular dynamics; MM-GBSA: Molecular Mechanics—Generalized Born Surface Area; ISGs: interferon-stimulated genes. ISG15: interferon-stimulated gene 15; TLR: Toll-like receptor; RSV: respiratory syncytial virus; OAT3: Organic Anion Transporter 3; RdRp: RNA-dependent RNA polymerase; IC^50^: half-maximal inhibitory concentration; GFP: green fluorescent protein; F protein: Fusion protein; mAb: monoclonal antibody.

**Table 4:** Research on immunization strategies.

Study (Author, Year)	Vaccine Type	Study Design/Model	Target Population	Key Findings	Conclusion
[135]	Live attenuated recombinant (rhMPV-Pa)	Phase I clinical trial	Adults, seropositive and seronegative children	Over-attenuated in children; low infectivity and immunogenicity	Trial discontinued due to insufficient infectivity in pediatric target group
[136]	Bivalent VLP (IVX-A12) +/– MF59^®^	Phase I clinical trial	Adults: 60–75 years	Safe and immunogenic, especially with higher doses	Continued development supported for older adults
[137]	Bivalent LAV (Metavac^®^-RSV)	Preclinical, BALB/c mice	Preclinical	Induced mucosal and systemic immunity; effective protection	Strong LAV candidate for mucosal immunization against RSV and hMPV
[137]	Prefusion-closed (UFC) trimers of F proteins	Biophysical studies and mouse models	Preclinical	Stable, potent neutralizing Ab response	Basis for next-gen prefusion F-based vaccines
[138]	VLP with HIV-1 Gag fusion (hMPV-F VLP)	Mouse models	Preclinical	Stronger immunogenicity in VLP-bound form, especially as prime	Gag-based VLPs are effective platforms for hMPV vaccines
[139]	Subunit + cpcQS-21 and TLR4 agonist	Mouse model	Preclinical	Comparable to commercial adjuvants, strong cellular/humoral responses	Plant-derived QS-21 is a viable, scalable adjuvant
[140]	mRNA-1653 (hMPV + PIV3)	Phase 1 clinical trial	Adults: 18–49 years	Good safety, elevated hMPV titers lasting 1 year	mRNA platform promising for hMPV vaccines
[141]	Stabilized Pre-F protein (dual-cleaved)	Structural studies + cotton rat challenge	Preclinical	High stability, expression, and strong neutralizing Ab	Strong candidate for next-gen subunit hMPV vaccine

rhMPV-Pa: recombinant human metapneumovirus (live attenuated); RSV: respiratory syncytial virus; hMPV: human metapneumovirus; VLP: virus-like particle; MF59^®^: proprietary adjuvant used in vaccine formulations; LAV: live attenuated vaccine; BALB/c: a strain of laboratory mice commonly used in research; UFC: uncleaved prefusion-closed; cpcQS-21: plant-cell-culture derived QS-21 (an adjuvant); TLR4: Toll-like receptor 4; mRNA: messenger RNA; PIV3: parainfluenza virus type 3; Pre-F: prefusion F protein (for RSV or hMPV).

## Data Availability

No new data were created or analyzed in this study. Data sharing is not applicable to this article.
